# The Paradox of Osteoporosis in Spondyloarthropathies

**DOI:** 10.31138/mjr.270924.poa

**Published:** 2024-12-31

**Authors:** Andreas Angelopoulos*, Ioannis Kouverianos*, Dimitrios Daoussis

**Affiliations:** 1 General Hospital of East Achaia, Aigio, Greece,; 2 Health Centre, Vonitsa, Greece,; 3 Department of Rheumatology, Patras University Hospital, University of Patras Medical School, Patras, Greece

**Keywords:** osteoporosis, psoriatic arthritis, axial spondylarthritis, spondyloarthropathies

## Abstract

**Introduction:**

Spondyloarthropathies (SpA) are a family of inflammatory disorders that affect the spine and peripheral joints. The most common representatives are axial Spondylarthritis (axSpA) and Psoriatic Arthritis (PsA). Despite the fact that SpA are characterised by new bone formation, paradoxically, total Bone Mineral Density (BMD) may be decreased.

**Methods:**

An electronic search was conducted on Medline in order to explore the prevalence, risk factors and pathophysiology of Osteoporosis (OP) in SpA patients.

**Results:**

The prevalence of OP globally is reported to be 18.3%. The prevalence of OP in Axial Spondylarthritis (axSpA) patients ranges from 11.7% to 34.4%, while in Psoriatic Arthritis (PsA) patients seems to be similar to the general population. Several factors have been proposed for the development of OP in SpA, such as corticosteroid use and physical inactivity. Moreover, systemic inflammation appears to participate in the pathophysiology of OP with inflammatory cytokines such as Tumour Necrosis Factor (TNF) and Interleukin (IL)-23/IL-17 potentially having a key role in the pathogenesis of bone loss.

**Discussion:**

The current literature points to the direction that OP is an established comorbidity in axSpA. Local or/and systemic inflammation is possibly the main pathway contributing to bone loss in axSpA patients. However, it remains unclear whether OP is an established comorbidity in PsA patients, as it seems that OP is a treatment-associated adverse event.

## INTRODUCTION

Spondyloarthropathies (SpA) refer to a group of inflammatory diseases with a strong genetic background affecting the axial spine and peripheral joints. Even though we do not know the triggering factors that induce local or/and systemic inflammation in SpA, several data point to the direction of genetic, environmental and mechanical factors as well as dysbiosis of the gut microbiota. Enthesitis is hallmark of these diseases and other extra-articular manifestations, such us uveitis and skin lesions are also common.^1^ In addition, SpA patients may have several comorbidities such as cardiovascular disease, depression, anxiety, inflammatory bowel disease as well as Osteoporosis (OP).^2,3^

OP is a systemic bone disease characterised by decreased bone mass, compromised bone strength and changes in bone architecture. Fragility fractures (fractures that occur spontaneously or on low trauma impact) are the main clinical manifestation.^4,5^ Normally, bone tissue homeostasis is achieved by the careful co-ordination of osteoclasts and osteoblasts. It is well-known that osteoblasts take part in the formation of new bone and osteoclasts reabsorb bone tissue. This continuous cycle is called the bone remodelling cycle, and it is critical for bone integrity. However, in OP there is an imbalance of formation and absorption of bone, leading to an increased osteoclastic activation. The activation of osteoclasts by inflammatory cytokines, such as Tumour Necrosis Factor (TNF), Interleukin (IL)-17A, and IL-6, contribute to bone loss. Alongside with interleukins, alterations in mechanical load contribute to a vicious cycle of OP on the trabecular bone of the vertebrae.^6–9^

It is well known that bone tissue is very sensitive to alterations of mechanical stress. Osteocytes can sense and respond to mechanical stimuli and this procedure is essential to bone homeostasis as it can lead to bone loss. In Axial Spondylarthritis (axSpA), syndesmophytes on the vertebrae are non-randomly distributed but are detected mainly in the posterolateral regions of the vertebrae. Local osteitis or/and systemic inflammation contribute to the formation of syndesmophytes in the cortical areas of the vertebrae. As new bone formation progresses, syndesmophytes can bridge themselves, leading to ankylosis of the spinal column. Upon completion of bone bridging, a reduction in mechanical loading on the trabecular bone occurs, leading to progressive bone loss. This endless cycle further promotes the bridging of the cortical areas of the vertebrae and thus further weakening trabecular bone. This phenomenon promotes osteoporosis and increases the risk of fragility fractures of the vertebrae.^7,9^

Paradoxically, in SpA two different processes regarding bone metabolism can occur at the same time. Not well known mechanical and/or inflammatory stimuli cause new bone formation in cortical areas of the vertebrae with the formation of syndesmophytes, leading to ankylosis. Simultaneously, a deterioration of bone micro-architecture is observed with loss of trabecular bone in vertebral bodies and subsequently, reduced bone mineral density (BMD) with increased fracture risk.^4,6,8,10^ These two opposing manifestations demonstrate the complex pathophysiology of SpA and pinpoint that more research is needed to understand the biological pathways of distributed bone remodeling in SpA. In this narrative review we focus on exploring the prevalence, risk factors and pathogenesis of OP in the context of the two most common types of SpA, axSpA and Psoriatic Arthritis (PsA).

## METHODS

We performed an electronic search on Medline using the following keywords: Osteoporosis, Psoriatic Arthritis, Axial Spondylarthritis, Spondyloarthropathies. Our main focus was to explore whether i) OP is more prevalent in SpA compared to the general population, ii) possible risk factors and finally iii) the pathophysiology of OP in SpA. Our research was carried out from March 2024 to July 2024. We only included relevant papers referring to the prevalence, risk factors and pathophysiology of OP in SpA and excluded articles not related to the objective of our review and not written in the English language.

## RESULTS

### Axial Spondylarthritis and Osteoporosis

AxSpA is known to be accompanied by a high prevalence of osteoporosis and fractures. OP in axSpA is multifactorial and may be caused by systematic inflammation and limited spinal mobility. The latter may diminish the frequency of outdoor exercises leading to reduced sun exposure and decreased vitamin D production. Physical inactivity or malabsorption if inflammatory bowel disease is present, are considered to be risk factors for the development of OP. Fragility fractures, such as fractures in the vertebrae and hips are a common outcome of osteoporosis in axSpA patients.^11^

Ramirez et al. recently conducted a systematic review and meta-analysis of observational studies. They report that approximately one in three axSpA patients may suffer from OP and one in four patients may suffer from fractures.^10^ According to a 2023 large retrospective matched cohort study, the incidence of OP in axSpA patients was higher and appeared earlier than the controlled group.^12^ Furthermore, the risk of OP and fractures in axSpA patients is higher in older patients and those with long course of the disease.^13^ Another study showed that axSpA patients developed hip fractures earlier and more often, compared to a matched cohort.^14^

The bone disease in axSpA is driven by inflammatory mediators, such as TNF and IL-17A, which activate osteoclasts. Furthermore, these cytokines influence the Wnt pathway by regulating molecules such as Dickkopf1 (Dkk1) and sclerostin. Dkk1, induced mainly by TNF, inhibits bone formation and enhances bone resorption in axSpA patients through blockade of the canonical Wnt pathway.^7,9,15^ Diarra et al. first reported the key role of Dkk1 as a regulator of joint remodeling in animal models of arthritis. They also provided data on functional Dkk1 levels (by using a functional ELISA assay that measures only Dkk1 bound to its relevant receptor) and found that axSpA patients have lower levels than healthy subjects.^16^ Daoussis et al. have found that axSpA patients have lower functional levels of Dkk1 despite higher circulating Dkk1 levels. These data alongside additional ex vivo experiments pointed to the direction that Dkk1 may be dysfunctional in axSpA patients, a finding with potential pathogenetic implications.^17–19^ Moreover, according to a 2016 meta-analysis, Dkk1 circulating levels were significantly higher in axSpA patients than in normal controls.^20^ A possible explanation of new bone formation in the cortical areas of the vertebrae may be the fact that TNF may not be able to significantly increase the production of Dkk1 or that the molecule itself is not functioning properly.^7^

Elevated levels of sclerostin in the serum of axSpA patients are associated with high disease activity and functional impairment in this group of people.^7,9,21^ However, a recent meta-analysis showed that there was no difference between sclerostin levels in axSpA patients and healthy controls.^22^ Gercik et al. reported that sclerostin levels were correlated negatively with both inflammation and osteoporosis.^23^ These discrepancies on sclerostin levels in axSpA patients could be attributed to the different stages of the disease activity.

The basic cytokines linked to axSpA are TNF and IL-17A. The involvement of these cytokines in the pathophysiology of the disease is also demonstrated by the fact that targeting these cytokines is a highly effective therapeutic strategy. Both TNF and IL-17A have been found to promote osteoclastogenesis and activate osteoclasts, leading to osteoporosis. TNF can activate osteoclasts by two pathways. Notably, TNF can directly induce osteoclast formation and can also promote osteoclastogenesis indirectly, through the Receptor Activator of Nuclear Factor kappa-Β Ligand (RANKL).^7,9^ It seems that TNF can stimulate the expression of RANKL in osteoblasts. Low BMD is prevalent in advanced axSpA patients, especially in the spine. The use of anti-TNF agents in the treatment of axSpA showed an improvement of lumbar spine and total hip BMD but the BMD of the femoral neck remained unchanged.^24,25^ However, a decrease in vertebral fractures has not been demonstrated.^26^ Furthermore, anti-TNF agents, as estimated by Positron Emission Tomography (PET) scan/Magnetic Resonance Imaging (MRI), have been shown to suppress the activity of osteoblasts in the spine and in the sacroiliac joints of axSpA patients.^27^ The effect of anti-TNF agents on new bone formation is the subject of a long-lasting debate; however, accumulating evidence indicating that long term use may retard radiographic progression.^28^ A 2020 systematic review and meta-analysis showed that the usage of anti-TNF treatment for more than 4 years was associated with delayed radiographic progression.^29^ Moreover, long term treatment with anti-TNF agents does not lead to an increase in the rate of new bone formation over 8 years in axSpA patients.^30^

**Figure 1.**
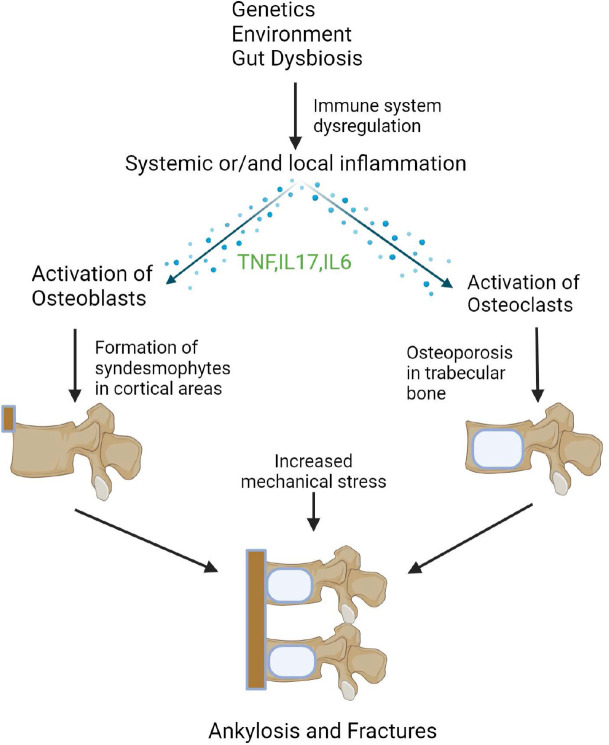
Pathogenesis of SpA.

IL-17A is a highly pro inflammatory cytokine produced not only by T-Helpers (Th-17) cells but by other cell types such as γδT cells and innate like lymphoid cells. IL-17A may directly promote osteoclastogenesis in axSpA patients.^7,9^ Moreover, IL-17A stimulates the expression of RANK on osteoclasts and consequently increases the sensitivity of RANK to bind RANKL, leading to the activation of osteoclasts.^7,18^ On the other hand, IL-17A involvement in osteoproliferation may be significant. Treatment with anti-IL-17A agents, such as secukinumab, showed that in 80% of axSpA patients, new bone formation in the vertebral column was inhibited, as assessed by radiographic imaging. Secukinumab proved to be safe and effective in reducing osteoporosis and suspending the formation of syndesmophytes in entheseal tissues, through a 5-year treatment.^31^ It is not well known how IL-17 is implicated in the process of new bone formation, but recent data indicate that IL-17A can suppress Dkk1 mRNA levels in human mesenchymal stem cells, a finding with potential pathogenetic implications.^32^

### Psoriatic Arthritis and Osteoporosis

Literature investigating the relationship between OP and PsA is ambiguous and often conflicting. It is well established that patients with PsA have many potential risk factors for OP including chronic systemic inflammation with increased levels of inflammatory cytokines such as IL-1, IL-6 and TNF-a.^33,34^ Other risk factors are long term corticosteroid use, low vitamin D, smoking, renal impairment and lack of physical exercise due to arthralgia or joint dysfunction.^4,33–35^ Furthermore, the IL-23/IL-17 pathway is a potential regulator of bone remodeling, as there is an overexpression of IL-23 in PsA which leads to an upregulation of IL-22 that may trigger osteoblastogenesis and enthesophytes formation in animal models.^36–38^ Some studies show that patients with PsA have lower volumetric body mass density (vBMD) compared to healthy individuals, while other report that PsA patients have similar, or even slightly higher BMDs compared to healthy controls.^33,36,39–43^ Nevertheless, four studies examining 442 postmenopausal, premenopausal women and men overall with PsA, did not find increased frequency of vertebral fractures (VF) or non-VF in PsA patients, which are the most serious complication of OP.^44–47^ Another study reported that serum calcium, phosphorus, alkaline phosphatase and bone turnover marker serum carboxy – terminal collagen crosslinks (CTX) did not differ in patients with PsA and controls.^4^ A Canadian cohort study found that the frequency of osteopenia and OP in PsA patients was 43.5% and 12.9% respectively, a prevalence similar to the general population. Meanwhile, recommendations and guidelines of Group for Research and Assessment of Psoriasis (GRAPPA) in 2021 conclude that OP is not a significant comorbidity in patients with PsA and their surveillance should be the same as for the general population.^48^

On the other hand, many studies support that there is a correlation between PsA and OP. For example, a cross-sectional study in the United States evaluated 28.765 people with PsA for a period of seven years, concluded that osteopenia, OP and fractures were more common in PsA patients compared with the control group.^34^ Two systematic reviews and meta-analyses indicated that PsA patients are in greater danger of developing fractures but did not find lower BMD or increased prevalence of OP in these patients. This could be explained by the fact that although fractures are linked to decreased bone strength, our means of evaluating bone quality such Dual-Energy X-Ray Absorptiometry (DEXA) do not give us information about microarchitecture, mineralisation, and marrow composition.^49,50^ The higher frequency of fractures is confirmed by other studies also reporting reduced bone density and increased falls in PsA patients.^51–53^ Additionally, in a Hungarian case control study and in a population-based case-controlled study in Israel, disease activity was an independent predictor for OP and fracture. Peripheral PsA is usually combined with more severe bone loss compared to axial disease.^35,54,55^ As for the gender, in patients with PsA, there is a higher prevalence of OP in women and patients over 50 years old.^56^ Lastly, a sample of 432,513 people from UK data-set was examined and the authors concluded that the effect of PsA in BMD/OP/fracture was secondary due to medication, and not causal. Treatment with ciclosporin has been revealed to affect the process of bone remodeling.^42^ Hitherto, data are conflicting, and more research is needed to explore the prevalence of OP in PsA, as well as whether there is a causal relationship between them.

## DISCUSSION

The high prevalence of OP and fractures in axSpA patients is demonstrated by several studies. Targeted therapies, such as anti-TNF and anti-IL-17 agents, show promising results in reducing inflammation and increasing spinal bone mass. It seems that anti-TNF and anti-IL-17 agents suspend osteoporosis but cannot prevent vertebral fractures. More research is needed in order to investigate whether there is a delay in the radiographic progression in patients treated with anti-TNF or anti-IL17 agents. Nevertheless, discontinuation of these agents contributes to rapid relapse of the disease and increased risk of fractures in the vertebrae.

The risk of OP in PsA patients remains unclear. Worse BMD is observed in patients with polyarticular disease and, as a result, these patients should be tested with a DEXA scan on a regular basis. Except for the aforementioned risk factors for OP, other characteristics seem to be protective against bone disease, such as obesity and the use of biologic therapies. Besides, GRAPPA recommendations and guidelines conclude that OP is not an important comorbidity in PsA patients, and their surveil-lance should be the same as for the general population. Many complex mechanisms are involved in pathogenesis of OP in SpA. Inhibition of one factor may not be enough to prevent the onset of OP in SpA patients. As a result, treatment should be multifocal and aim at multiple targets in order to ensure the best outcome for these patients.

In conclusion, SpA have a major impact on patients’ daily life, causing reduced mobility, joint pain and diminished quality of life. SpA are usually accompanied with several comorbidities worsening patients’ functionality. OP has been shown to be a significant comorbidity in axSpA patients with a high prevalence of fragility fractures, unlike PsA where there is disagreement for the role of OP in the disease.
